# Supplementary figures and images for: Iron nanoparticle-labeled murine mesenchymal stromal cells in an osteoarthritic model persists and suggests anti-inflammatory mechanism of action
Source: PLoS One. 2019 Dec 3;14(12):e0214107. doi: 10.1371/journal.pone.0214107 (PMC6890235; doi:10.1371/journal.pone.0214107)

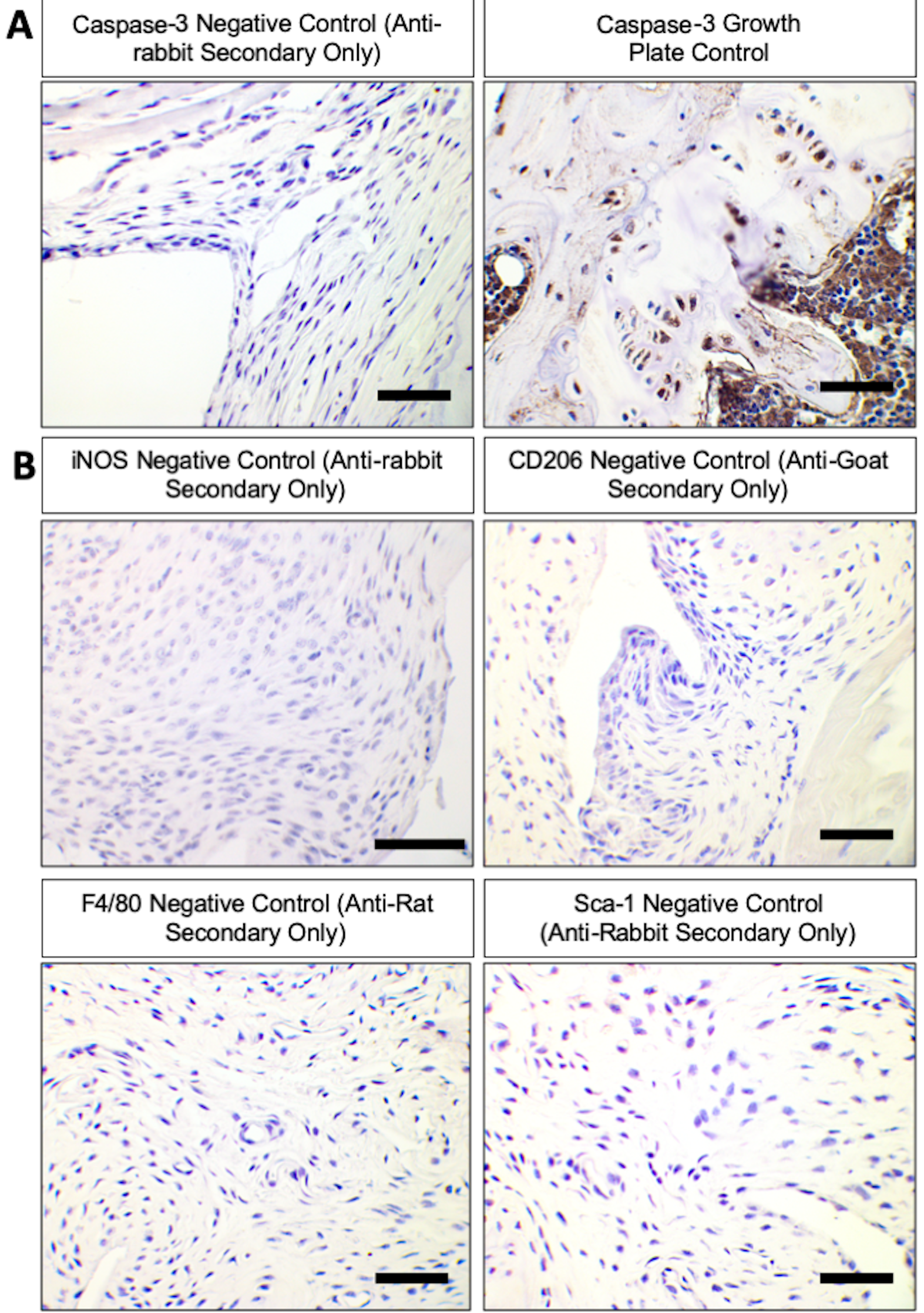

Supplement: S1 Fig — (A) Controls for Rabbit-Anti-Mouse cleaved Caspase-3 antibody. (Left) Synovial tissue was stained with anti-rabbit secondary only, counterstained with hematoxylin. (Right) Caspase-3 displayed specific immunostain in growth plate control, as hypertrophic chondrocytes undergo apoptosis within the growth plate. (B) No primary Antibody negative control for and iNOS (Top left) CD206 (Top right), F4/80 (Lower left) and Sca-1 (lower right) respectively, using anti-goat secondary and anti-rat secondary antibody. Scalebars are 100 μm. (TIFF) [file pone.0214107.s001.tiff]

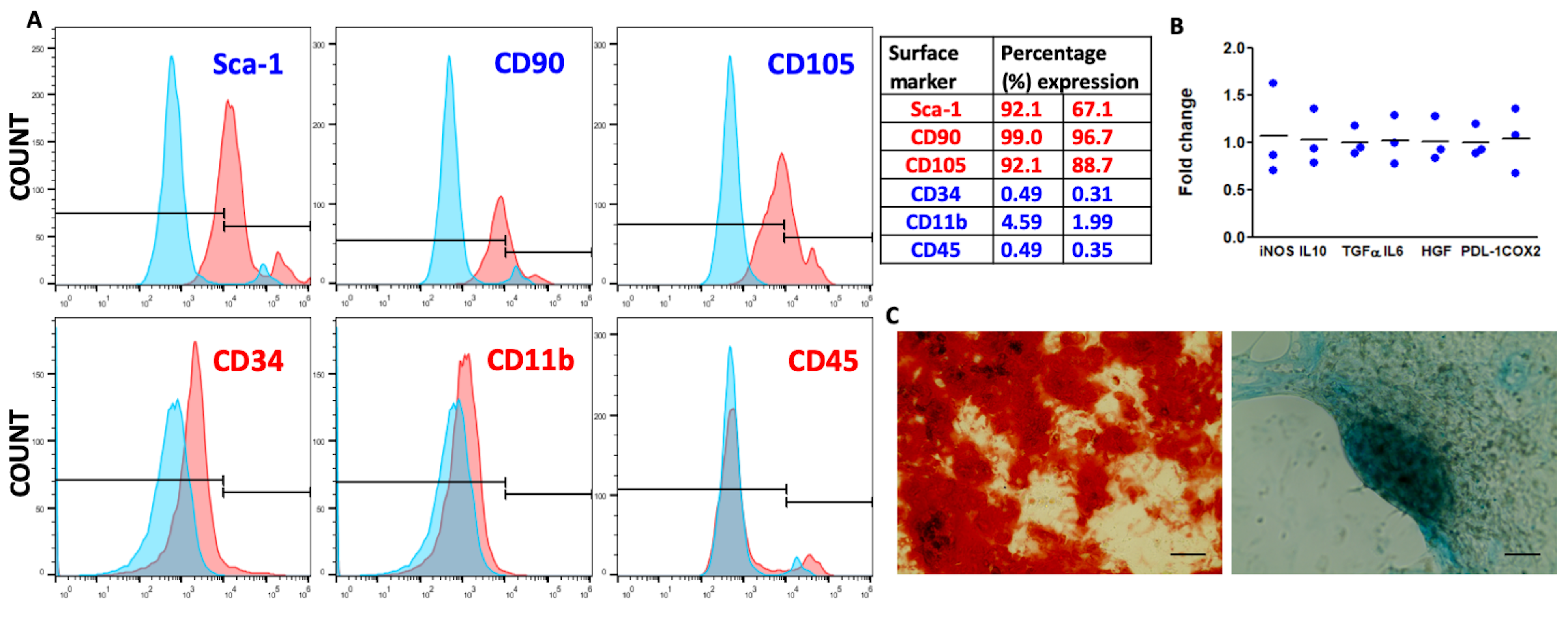

Supplement: S2 Fig — (A) Immuophenotyping of murine MSCs. Blue histograms represent unstained cells and red overlay histograms are for positive MSC markers (Sca-1, CD90, CD105) and negative hematopoietic markers (CD45, CD11b, CD34). MSC associated (shown in red) and non-associated markers (shown in blue). Table (right) indicates the percent expression of each marker for both cell populations. (B) Differential gene expression analysis of MSC. Data shown are fold change relative to housekeeping gene mouse beta-2-microglobulin (mB2M) as analyzed by qPCR. (C) Osteogenic differentiation and chondrogenic differentiation of MSCs using Human Mesenchymal Stem Cell (hMSC) Differentiation Kit (ThermoFisher). Images were taken using EVOS microscope at 10X magnification (scale bar:100 μm). (TIFF) [file pone.0214107.s002.tiff]

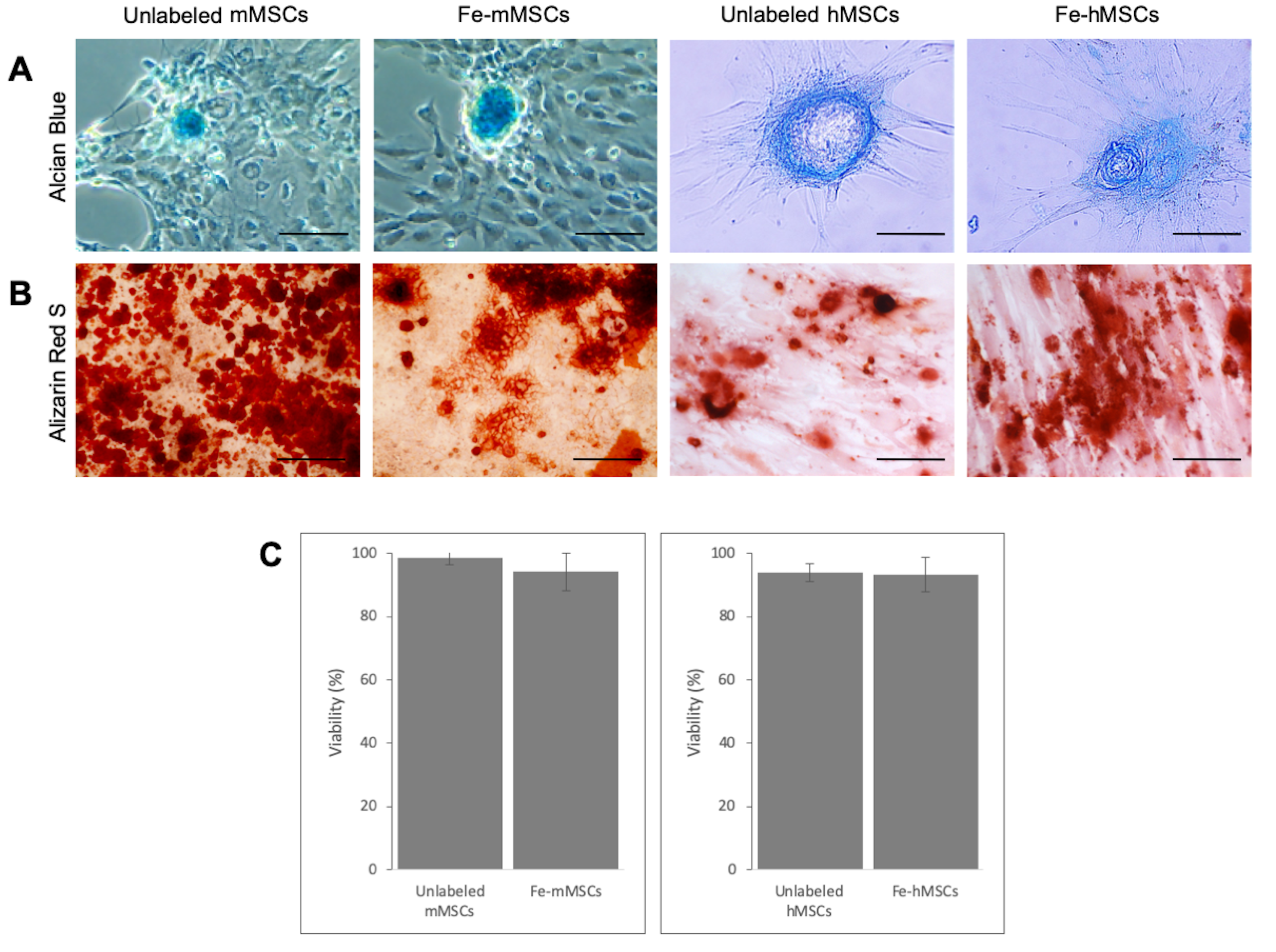

Supplement: S3 Fig — (A) Chondrogenesis stained with Alcian Blue and (B) Osteogenesis stained with Alizarin Red S. Scalebar = 25 μm; mMSC: murine MSC; hMSC: human MSC. C. No viability differences in ferumoxytol-labeled murine and human MSCs vs. unlabeled murine and human MSCs. Viability assessed by Trypan Blue exclusion assay expressed as percentages. murine MSCs (n = 4). human MSCs (n = 3). (TIFF) [file pone.0214107.s003.tiff]
