# Supplementary material for: Iron nanoparticle-labeled murine mesenchymal stromal cells in an osteoarthritic model persists and suggests anti-inflammatory mechanism of action
Source: PLoS One. 2019 Dec 3;14(12):e0214107. doi: 10.1371/journal.pone.0214107 (PMC6890235; doi:10.1371/journal.pone.0214107)
Supplement: S1 Table — (DOCX) [file pone.0214107.s004.docx]

| *Gene/Primer* | *Sequence* |
| --- | --- |
| *B2M-F* | 5’-CATGGCTCGCTCGGTGACC-3’ |
| *B2M-R* | 5’-AATGTGAGGCGGGTGGAACTG-3’ |
| *iNOS-F* | 5’-CCCTTCAATGGTTGGTACATGG-3’ |
| *iNOS-R* | 5’-ACATTGATCTCCGTGACAGCC-3’ |
| *IL10-F* | 5’-TAACTGCACCCACTTCCCAG-3’ |
| *IL10-R* | 5’-TTGTCCAGCTGGTCCTTTGT-3’ |
| *TGFβ1-F* | 5’-AGCTGCGCTTGCAGAGATTA-3’ |
| *TGFβ1-R* | 5’-TGCCGTACAACTCCAGTGAC-3’ |
| *IL6-F* | 5’-ATGGATGCTACCAAACTGGAT-3’ |
| *IL6-R* | 5’-TGAAGGACTCTGGCTTTGTCT-3’ |
| *HGF-F* | 5’-ATGTGGGGGACCAAACTTCTG-3’ |
| *HGF-R* | 5’-GGATGGCGACATGAAGCAG-3’ |
| *PDL-1-F (or CD74)* | 5’-AGTATGGCAGCAACGTCACG-3’ |
| *PDL-1-R (or CD74)* | 5’-TCCTTTTCCCAGTACACCACTA-3’ |
| *COX2-F (or PTGS2)* | 5’-TTCCAATCCATGTCAAAACCGT-3’ |
| *COX2-R (or PTGS2)* | 5’-AGTCCGGGTACAGTCACACTT-3’ |
