# Supplementary material for: Iron nanoparticle-labeled murine mesenchymal stromal cells in an osteoarthritic model persists and suggests anti-inflammatory mechanism of action
Source: PLoS One. 2019 Dec 3;14(12):e0214107. doi: 10.1371/journal.pone.0214107 (PMC6890235; doi:10.1371/journal.pone.0214107)
Supplement: S2 Table — (DOCX) [file pone.0214107.s005.docx]

| **Name** | **Clone Number** |
| --- | --- |
| PerCP/Cy5.5 anti-mouse I-A/I-E | M5/114.15.2 |
| Rat mAb to Sca1/Ly6A/E [D7](FITC) | D7 |
| APC anti-mouse CD11b | M1/70 |
| PE/Dazzle™ 594 anti-mouse/human CD45R/B220 | RA3-6B2 |
| PE anti-mouse/rat CD29 | HMβ1-1 |
| PE labeled anti-mouse CD34 (RAM34) | RAM34 (RUO) |
| PE conjugated anti-mouse CD105(endoglin) | MJ7/18 |
| FITC rat IgG2a, κ isotype standard(R35-95) | R35-95 |
| APC mouse IgG1, κ isotype CTRL | MOPC-21 |
| APC anti-human CD90(Thy1) | 5.00E+10 |
| FITC anti-human IgG | G18-145 |
