# Supplementary material for: Iron nanoparticle-labeled murine mesenchymal stromal cells in an osteoarthritic model persists and suggests anti-inflammatory mechanism of action
Source: PLoS One. 2019 Dec 3;14(12):e0214107. doi: 10.1371/journal.pone.0214107 (PMC6890235; doi:10.1371/journal.pone.0214107)
Supplement: S3 Table — (DOCX) [file pone.0214107.s006.docx]

| *Biochemical Parameter (Range and units)* | *DMM*  *DiR+Fe-MSC*  *(2 weeks)* | *DMM*  *DiR MSC*  *(2 weeks)* | *DMM*  *DiR+Fe-MSC*  *(4 weeks)* | *DMM*  *DiR MSC*  *(4 weeks)* |
| --- | --- | --- | --- | --- |
| *Albumin (21-34 g/L)* | 23.6 ± 4.34 | 21.6 ± 5.55 | 25.6 ± 6.54 | 25 ± 11.60 |
| *Alakaline Phosphatase (28-94 U/L)* | 44.8 ± 8.79 | 52.4 ± 12.20 | 36.8 ± 18.95 | 30 ± 13.95 |
| *Alanine Transaminase (28-184 U/L)* | 27.2 ± 6.72 | 51.6 ± 33.27 | 24.4 ± 7.80 | 56 ± 45.46 |
| *Amylase (U/L)* | 596 ± 79.94 | 879.2 ± 511.43 | 656.4 ± 169.94 | 607 ± 158.11 |
| *TBIL (µmol/L)* | 7.2 ± 1.10 | 7.2 ± 1.10 | 7.6 ± 0.89 | 10 ± 4.00 |
| *Blood Urea Nitrogen (12.1-20.5 mmol/L)* | 6.56 ± 1.10 | 5.92 ± 1.94 | 5.2 ± 1.24 | 7.25 ± 0.81 |
| *Calcium (2.77-3.02 mmol/L)* | 2.228 ± 0.12 | 2.236 ± 0.14 | 2.08 ± 0.43 | 2.395 ± 0.44 |
| *Phosphorus (2.88-3.62 mmol/L)* | 3.236 ± 1.28 | 3.708 ± 1.06 | 2.988 ± 0.19 | 3.455 ± 0.94 |
| *Creatinine (µmol/L)* | 28.8 ± 18.20 | 16.4 ± 16.02 | 48.8 ± 10.45 | 42 ± 12.00 |
| *Glucose (9.7-18.6 mmol/L)* | 7.88 ± 5.08 | 8.24 ± 5.08 | 9.12 ± 2.88 | 8.25 ± 2.51 |
| *Sodium (143-150 mmol/L)* | 304.4 ± 10.14 | 307.6 ± 8.99 | 292.4 ± 3.29 | 293.5 ± 2.52 |
| *Potassium(3.8-10.0 mmol/L)* | 7.04 ± 2.81 | 8.56 ± 5.04 | 6.08 ± 1.29 | 8.1 ± 2.52 |
| *Total Protein (g/L)* | 49.2 ± 1.10 | 48 ± 3.46 | 46 ± 9.38 | 50 ± 4.9 |
| *Globulin (18-82 g/L)* | 20.4 ± 11.70 | 9.2 ± 12.62 | 18.8 ± 11.37 | 15.5 ± 10.5 |
