# Supplementary material for: Iron nanoparticle-labeled murine mesenchymal stromal cells in an osteoarthritic model persists and suggests anti-inflammatory mechanism of action
Source: PLoS One. 2019 Dec 3;14(12):e0214107. doi: 10.1371/journal.pone.0214107 (PMC6890235; doi:10.1371/journal.pone.0214107)
Supplement: S4 Table — The report shows that Fe-MSCs are safe as assessed by gross pathology of heart and spleen. n/s: No significant findings; H*: The majority of the myocardium appears normal. There is one region of endocardium that has a small amount of fibrin deposition. Duration: subacute; Distribution: focal; Severity: moderate; S*: There are a few areas of decreased density in the periphery of the red pulp. The marginal zones also appear moderately decreased. (DOCX) [file pone.0214107.s007.docx]

| *Cell type* | *Timepoint* | *Heart* | *Spleen* |
| --- | --- | --- | --- |
| DiR+MSC | 2 weeks | n/s | n/s |
| DiR+MSC | 2 weeks | n/s | n/s |
| DiR+MSC | 2 weeks | n/s | n/s |
| DiR+MSC | 2 weeks | n/s | n/s |
| DiR+MSC | 4 weeks | n/s | n/s |
| DiR+Fe-MSC | 2 weeks | n/s | n/s |
| DiR+Fe-MSC | 2 weeks | n/s | n/s |
| DiR+Fe-MSC | 2 weeks | n/s | n/s |
| DiR+Fe-MSC | 2 weeks | n/s | **S*** |
| DiR+Fe-MSC | 2 weeks | n/s | n/s |
| DiR+Fe-MSC | 2 weeks | n/s | n/s |
| DiR+Fe-MSC | 2 weeks | n/s | n/s |
| DiR+Fe-MSC | 2 weeks | n/s | n/s |
| DiR+Fe-MSC | 2 weeks | **H*** | n/s |
| DiR+Fe-MSC | 4 weeks | n/s | n/s |
| DiR+Fe-MSC | 4 weeks | n/s | n/s |
| DiR+Fe-MSC | 4 weeks | n/s | **S*** |
| DiR+Fe-MSC | 4 weeks | n/s | n/s |
| DiR+Fe-MSC | 4 weeks | n/s | **S*** |
